# Supplementary figures and images for: Are carbon emissions trading and green financial instruments synergistic? -Comprehensive quantitative research based on content analysis
Source: PLoS One. 2024 Mar 7;19(3):e0298601. doi: 10.1371/journal.pone.0298601 (PMC10919601; doi:10.1371/journal.pone.0298601)

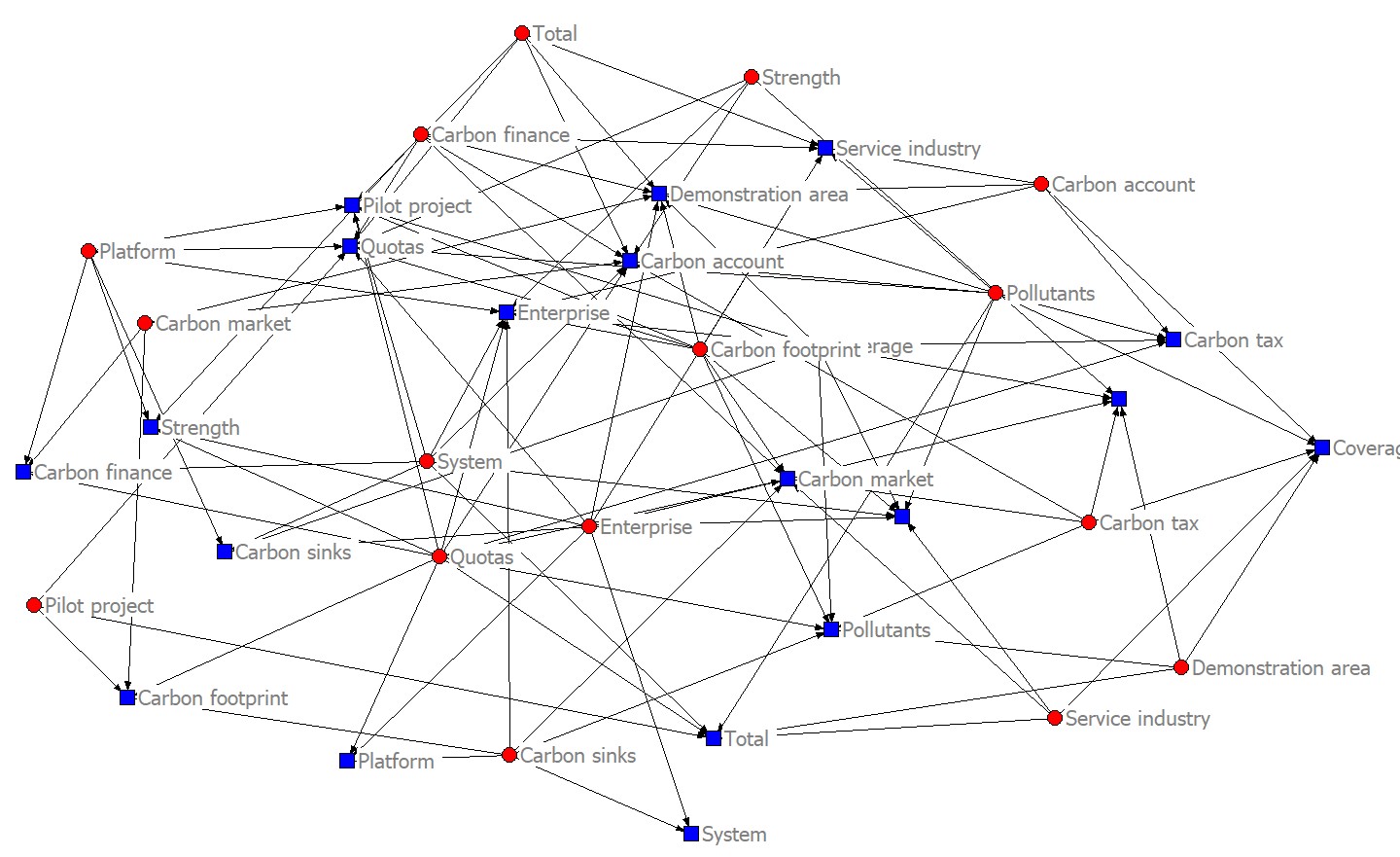

Supplement: S1 Data — (ZIP) [file pone.0298601.s001.zip › Data/policy network.jpg]

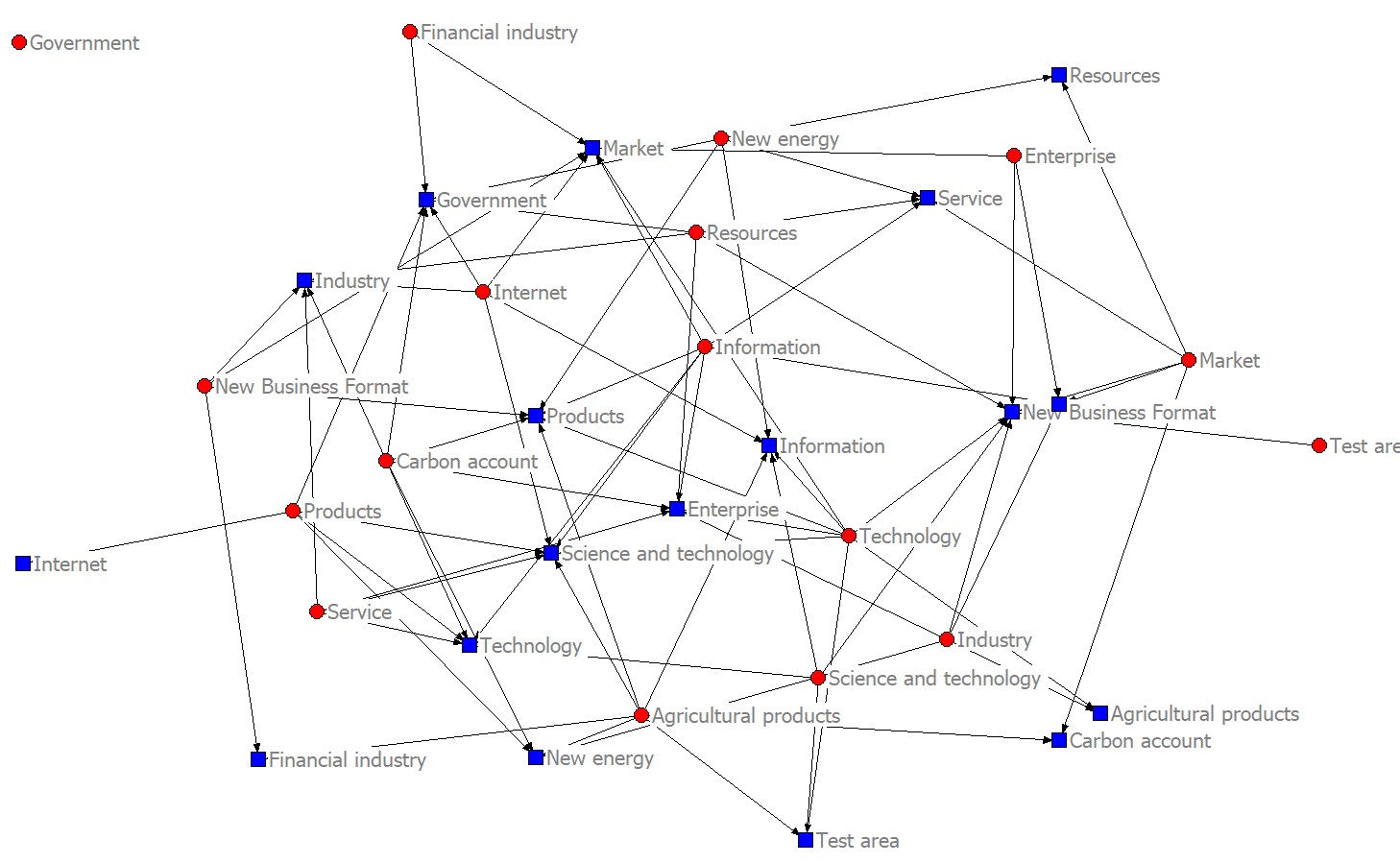

Supplement: S1 Data — (ZIP) [file pone.0298601.s001.zip › Data/policy2.jpg]
