# Supplementary material for: Are carbon emissions trading and green financial instruments synergistic? -Comprehensive quantitative research based on content analysis
Source: PLoS One. 2024 Mar 7;19(3):e0298601. doi: 10.1371/journal.pone.0298601 (PMC10919601; doi:10.1371/journal.pone.0298601)
Supplement: Attachment — Submitted filename: Responses to Reviewers(1).docx [file pone.0298601.s002.docx]

**Response to Reviewers**

Thank you very much for the revision suggestions of the study, which have allowed us to gain a lot, and deepened more thinking to better improve our study. We hope that our revisions can make the quality of the article better. If there are any inappropriate revisions, we will continue to work hard. Hope to have the opportunity to be published. Thanks again! The following is the specific modification part and we mark the changes in red:

Reviewer #1: This paper makes word frequency statistics according to the policy system, and summarizes the core objectives and tools of green financial policy and carbon emission trading policy. For the classification of policy tools, according to the characteristics of green finance policy and carbon emission reduction policy, this paper divides policy tools into ' command control type ', ' market incentive type ' and ' public participation type '.Based on the existing policy network, the carbon emission reduction policy system is constructed, and the future direction of using green finance and carbon emission trading policy to guide the development of coordinated emission reduction is analyzed. The article is innovative. In order to improve the quality of the article, we put forward the following suggestions.

1. The theoretical analyses in abstract of the article is overloaded, and it is recommended that some of them be reorganised or included in the introduction.

Thanks for the valuable advice from the experts. We organize the abstract part of the article and explain the research purpose, methods, results and main contributions of the article. At the same time, part of the content is placed in the introduction. The specific modifications are as follows:

Coordinating policies is an essential guarantee for carbon emission reduction and sustainable development. Based on the theoretical framework of the policy paradigm, we quantitatively analyze 266 policy documents on promoting carbon emission trading and green financial policies from 2011 to 2022 using the content analysis research method. Based on the matching network of "policy objectives-policy tools," we analyze the synergistic characteristics of carbon emission trading policies and green financial policies in promoting carbon emission reduction targets and reveal the matching mode of "objectives-tools" of green financial policies by using social network analysis. It is found that, first, from the perspective of policy objectives, the main policy objectives of carbon emissions trading are to promote green innovation of enterprises, and the main policy objectives of green finance are to promote green development, which reflects the consistency and endogenous motivation of policy objectives. Secondly, command-control and market incentive policy tools are the main policy tools in the structure of policy tools. The proportion of public participation policy tools is small, and there is a structural asymmetry. Third, carbon emissions trading tools focus on supervision, adjustment, and platform construction. The green financial policy tools have the characteristics of guidance, public welfare, and externality. The two constitute a complementary, embedded, and integrated ' double synergy ' carbon emission reduction policy. Based on this, this paper puts forward some suggestions to promote policy coordination and provides a reference for China to achieve the dual carbon goal.

2. Literature review. The analysis of literature review is not systematic enough. It is suggested to add the latest research results and point out the gaps in the existing research, so as to show the innovation and necessity of this paper.

Thanks for the valuable advice from the experts. We add the latest research results to the literature review, so as to summarize the existing research results and findings, and find out the shortcomings of the existing research, which better reflects the innovation of this research. The specific modifications are as follows:

literature review

Carbon trading policy

...

Green financial policy

...

The green financial policy affects and increases the debt financing cost of heavily polluting enterprises. Therefore, the green financial system should improve further [30]. Therefore, the supporting policies, measures, and development strategies of green finance are major mechanism innovations and policy tools to promote the transformation and development of the low-carbon economy in China.

Policy mix

...

Through a combination of policy tools, energy efficiency policies are expected to play a vital role in achieving EU energy goals, especially in reducing energy demand and CO2 emissions. Transaction costs are integrated into analyzing pollution problems and policies to overcome pollution problems. The policy mix may help correct multiple strengthening failures of private governance structures, such as pollution externalities and technology spillovers.

...

Intergovernmental cooperation is a cooperative relationship between governments at all levels to formulate and implement policies or provide services [40,41]. The cooperation between governments at all levels is more reflected as a kind of mutual synergy, which can better optimize the efficiency of regional resource allocation and achieve the goal of multi-subject win-win [42].

3. Further beautiful charts to make it more standardized. In particular, Figure 2 and Figure 3.There are many tables in this paper, and it is recommended to set the table as a three-line table and improve the format of the table.

Thanks for the valuable advice from the experts. We have revised and embellished the charts of the full text, and modified the format of the full text according to the style and requirements of the PLos One journal. See the text for specific modifications.

4. Research methods and data processing. I suggest adding a key time node table for some policies. Analyze policies at different levels to highlight intergovernmental relations.

Thanks for the valuable advice from the experts. We increase the time node, policy keywords and network analysis of the policy to better quantify the policy text. The specific modifications are as follows:

**Integrated frame of policy analysis**

Based on the constructed ' policy objectives-policy tools ' dual policy network structure, we analyze the carbon emission trading and green financial policy texts from four dimensions, as shown in Fig 1.

Policy aim

Policy instrument

Publishing authority hierarchy

Green innovation

Green development

Carbon emission reduction

Quota allocation

Public participation

Pilot participation

……

……

Policy time

**Fig 1.** Carbon emissions trading and green financial policy collaborative research framework

Source: Self-painted by the author

The regional spatial hierarchy dimension divides the policy text into provincial spatial levels according to the administrative level of the policy issuing unit. The policy release time covers 2011-2022, reflecting the changes in the number of policies and themes over time. The policy content dimension is based on the specific policy content in the text.

**Characteristics of policy text**

**...**

**Time dimension analysis**

From 2011 to 2021, the total number of relevant policies generally showed a continuous upward trend, and the comparison chart of the number of central and local policy releases is shown in Fig 2. From the overall level of coordination between carbon emissions trading and green finance, the "Notice of the National Development and Reform Commission on the Pilot Work of Carbon Emissions Trading" gives a top-level design plan for carbon trading. In 2012, the China Banking Regulatory Commission issued No.4 to formulate "Green Credit Guidelines" to guide the development of green finance. As a result, an overall guiding policy for carbon emission trading and green finance to promote green development has been gradually formed. From the provincial level, the annual number of policy texts in the carbon trading pilot market is large, and the difference in changes is small. Continue to pay attention to the construction and improvement of the carbon emission trading market. A comparison of the number of central and local policy releases is shown in Fig 2.

|  | Center | Local |
| --- | --- | --- |
| 2011 | 2 | 1 |
| 2012 | 3 | 6 |
| 2013 | 5 | 16 |
| 2014 | 3 | 9 |
| 2015 | 5 | 10 |
| 2016 | 12 | 16 |
| 2017 | 9 | 24 |
| 2018 | 10 | 21 |
| 2019 | 8 | 22 |
| 2020 | 8 | 36 |
| 2021 | 13 | 27 |

**Fig** 2. Comparison of the number of central and local policy releases

From the perspective of the main body, the number of local policy releases is higher than that of the central policy. In view of the continuous construction and improvement of the green financial reform and innovation pilot area and the carbon emission trading pilot market, local governments need to constantly formulate relevant policies to guide the development of the market. Different regions can also use the opportunity of coordinated development of carbon emission trading and green finance to improve their own green development level.

**Policy focus**

The content features of policy texts are mainly expressed by their complex semantic features. Word frequency features can help understand the policy content and high-frequency words can represent the policy focus. 266 policy documents are counted according to the policy system, and word clouds are drawn, as shown in Figures 3 and 4

....

The high-frequency vocabulary is divided into policy objects and policy behaviors, and the word frequency statistics of promoting carbon emission trading policies are carried out according to two words and three words respectively, as shown in Table 2. The core words and network connections of carbon trading policy content are shown in Fig 5.

**Fig** 5. Carbon trading policy content core words and network connection diagram

In the vocabulary of policy objects of carbon emission trading, the top three words are "mechanism," "pilot," and "total amount," and the top three words are "pollutant," "coverage," and "service industry"; In the vocabulary of policy behavior, the top three words are "double control," "development" and "coverage," and the top three words are "carbon trading," "carbon emission" and "carbon peak". Divide high-frequency vocabulary into policy objects and behaviors, and promote green financial policies to make word frequency statistics according to two-word and three-word, respectively, as shown in Table 2. The core words and network links of green financial policy content are shown in Fig 6.

**Fig 6**. Green financial policy content core words and network connection diagram

In the vocabulary of policy objects of green finance, the top three words are "technology," "enterprise," and "government," and the top three words are "financial industry," "new energy," and "experimental area"; In the vocabulary of policy behavior, the top three words are "innovation," "development" and "service"; The top three words are "digitalization," "informationization" and "diversification."

5. Results and Analysis. I propose to add a discussion section, move some of the analysis in the results to the discussion, and compare the results of the article with the existing literature to better illustrate the innovation and contribution of this article.

Thanks for the valuable advice from the experts. We added the discussion section, the specific modifications are as follows:

**Discussion**

**Policy synergy analysis**

**...**

**Qualitative analysis of policy texts**

**...**

**Challenges in the collaborative process**

From the public participation policy tools perspective, the two policy systems actively attract more people to participate in activities such as carbon neutralization and emission reduction. The policy tools used include implementing a personal investment system, establishing a public access system, promoting green projects, replicating and popularizing the experience of first-in-first-trial, and encouraging individuals to participate in green finance and carbon trading. Under the carbon emission trading policy, there is a precedent for individuals to enter the market. As an innovative carbon finance model, carbon inclusive will transform individuals' low-carbon behavior into economic value and activate market liquidity. As an energy-saving and emission-reduction behavior, carbon inclusiveness increases the actual demand for carbon sink projects. Green finance policy is issued to individual investors, broadening the channels for the public to participate in green investment and expanding green finance's social effects. Both can strengthen the public's awareness of green responsibility investment to protect the environment and support green development. Analyzing the synergy of policy tools under different systems shows that carbon emission trading policies are characterized by supervision, adjustment, and platform. In contrast, green financial policy tools are characterized by guidance, public welfare, and externality, constituting a "dual synergy" carbon emission reduction policy that is complementary, embedded, and integrated. Based on the perspective of policy tool goal, we find the following problems and challenges in the process of policy synergy:

First, the choice of policy tools is diversified, but there is a significant structural imbalance. The low degree of adaptation between tools and goals restricts the effectiveness of policies, which will lead to a certain degree of deviation between realistic goals and policy expectations, and cannot meet the policy needs of the current market-driven stage. Green financial policy has a solid positive external effect in supporting enterprises' green development. To a certain extent, green financial products have the characteristics of public goods, and their essence is to protect and improve the ecological environment. Green financial products and services will incur costs, while consumers will not incur any costs when enjoying the benefits of ecological environment improvement. Therefore, there may be a need for more supply. This is consistent with Volz et al.'s (2015) and Soundarrajan & Vivek (2016) research [50,.51] There are structural imbalances such as the overuse of command tools, the relative underuse of symbol and exhortation tools, and the serious absence of capacity-building tools, incentive tools, and system change tools. We can further define the carbon intensity based on the relevant theories of new institutional economics, stipulate the total amount of carbon intensity in the assets of all financial institutions, and issue licenses for financial institutions. By establishing a perfect standard system, information disclosure, and other mechanisms, we can solve the dilemma of green financial policy through marketization.

Second, the government's attention configuration is more concentrated. Green finance has a 'free rider 'behavior. From the perspective of government decision-making, government decision-makers take green innovation and green development as policy objectives and draw a blueprint for the promotion of dual-carbon goals and work, providing direction for action. However, it should be noted that green projects usually have low returns and long payback periods and need additional certification for green projects. The financial "profit-seeking" feature will further strengthen financial institutions' "free rider" behavior in developing green finance. We should pay attention to the matching of policy objectives and tools, so as to 'improve quality and efficiency' while taking into account the comprehensive governance process of supervising high-energy-consuming enterprises, supporting green project construction, maintaining the healthy development of the market, and establishing a coordinated working mechanism. This further extends the research content of Guo & Tan (2023) and Dunlap & Laratte (2022) [52,53]. Therefore, we should prioritize making up for market failure, strengthening policy support for technological innovation and industrial upgrading by coordinating carbon trading and green financial policies, and improving "free rider behavior."

Third, enhance policy orientation. The characteristics of policy tool selection between different objectives are similar, which can easily lead to policy failure. Some policy tools with a high frequency of application are not clear enough, resulting in uncertainty in the implementation effect. For example, market-based policy tools are mostly supported, strengthened, and encouraged in expression, and there are no clear implementation provisions such as support mechanisms, forms, and channels, resulting in uncertainty in the degree and effect of implementation. Carbon finance is an essential product that reflects the synergy between green finance and carbon emission trading. It is still in the stage of product development, and there are risks such as high cost and abnormal fluctuation of carbon price. Some problems in carbon finance, such as complex technology, long cycle, low profit, high policy risk, asymmetric information, lead to insufficient motivation for carbon finance to a certain extent. Green finance is still dominated by traditional project financing and green credit, and its product structure and service mode are single. This complements the research contents of Zhou & Li (2019) and Simon et al. (2012) [15,54]. We can further innovate green financial products and services, implement environmental marketization means, improve the pilot design, operation, and performance of carbon emissions trading system, and seek innovative breakthroughs in project construction and management.

Reviewer #2:

This paper studies the optimization of China's carbon emission trading and green finance policies. The applied data are reliable, the research methods are feasible, the research conclusions are enlightening, and three optimization strategies are obtained. However, the optimization strategy is too macro, and it is hoped that the author can make more elaboration on this aspect of "improving the content of the policy system, improving the coordination goal of the policy system, and promoting the optimization and balance of policy tools", so as to improve the pertinence and operability of the countermeasures. At the same time, the format and language of the paper need to be refined and modified to achieve the integration of academic and native English.

Thanks for the valuable advice from the experts. We will make more elaborations on ' improving the content of the policy system, improving the coordination objectives of the policy system, and promoting the optimal balance of policy tools ', so as to improve the pertinence and operability of the countermeasures. The specific modifications are as follows :

**Management enlightenment**

Based on the synergistic characteristics of carbon emission reduction policies, we put forward the following policy suggestions. Based on the quantitative analysis of policy content, we believe that China should optimize the carbon emission trading mechanism and green financial policy tools from the following three aspects, and play a synergistic effect to achieve the dual carbon goal:

First, moderately increase the proportion of command-and-control policy tools. ...For market incentive environmental policy tools, we should continue to carry out technology promotion, government procurement, pilot/demonstration projects, etc., and accelerate the applicability promotion of zero-carbon building technology. For example, in the field of transportation, the use of market incentive policy tools should be improved, such as stimulating the market demand for new energy vehicles and helping the market-driven transformation of the new energy vehicle industry.

Second, improve the policy system of target coordination. Maintain the cohesion of different types of environmental policies. For example, the industrial sector should comprehensively use the financial subsidies, science and technology investment, and incentive mechanisms of the sub-categories of market-based incentive policy tools, the industrial layout, structural adjustment, tax incentives, financial support, and access rules of the sub-categories of command-and-control policy tools, and the incentives of public participation-based policies to accelerate the improvement of policy tools in different fields. We should actively promote the application of clean technology, formulate stricter control rules and access rules to promote energy efficiency, and formulate multi-type environmental policies of price, tax, and financial support. ...

Third, promote the optimization and balance of policy tools. Improve the accuracy and enforcement of various types of policy tools to enhance the breadth and threshold standards for the application of access rule policy tools. Formulate clear support policies and preferential tax policies, stimulate market demand, and effectively play a role in promoting green development.

At the same time, we refine and modify the format and language of the paper, and ask native English speakers to polish the language to achieve the integration of academic English and native English.
